# Prediabetes and Cardiovascular Parameters in Obese Children and Adolescents

**DOI:** 10.4274/jcrpe.2248

**Published:** 2016-03-01

**Authors:** Beray Selver Eklioğlu, Mehmet Emre Atabek, Nesibe Akyürek, Hayrullah Alp

**Affiliations:** 1 Necmettin Erbakan University Faculty of Medicine, Department of Pediatrics, Division of Pediatric Endocrinology and Diabetes, Konya, Turkey; 2 Konya Training and Research Hospital, Clinic of Pediatric Endocrinology and Diabetes, Konya, Turkey; 3 Malatya State Hospital, Clinic of Pediatric Cardiology, Malatya, Turkey

**Keywords:** obesity, prediabetes, children, adolescent

## Abstract

**Objective:**

In this study, our aim was to determine cardiovascular risk and cardiac function in prediabetic obese children and adolescents.

**Methods:**

The study was conducted on 198 obese children and adolescents 6-18 years of age. Anthropometric measurements, blood pressure measurements, oral glucose tolerance test, lipid profile, and HbA1c levels of patients were assessed. Prediabetes was defined according to American Diabetes Association criteria. Left ventricular mass index (LVMi), carotid intima-media thickness (c-IMT), and tissue Doppler measurements records were used.

**Results:**

LVMi was found to be significantly higher in the prediabetes group (p=0.03). There were no statistically significant differences in right ventricular tissue Doppler measurements between the prediabetic and non-prediabetic groups. Left ventricular tissue Doppler measurements were significantly higher in the prediabetes group: LVEEM (left ventricular E/e ratio) (p=0.04); LVEM (left ventricular myocardial velocity cm/s) (p=0.035). LVMi was found to positively correlate with triglyceride level, diastolic blood pressure, waist circumference, body weight standard deviation score and to negatively correlate with high-density lipoprotein cholesterol (p=0.043, r=0.15; p=0.039, r=0.15; p=0.025, r=0.17; p=0.009, r=0.19; p=0.038, r=-0.15, respectively). LVEM was correlated with glucose (p=0.046, r=0.15) and LVEEM was correlated with systolic blood pressure (p=0.035, r=0.15). In linear regression analysis for clinical cardiovascular risk factors, fasting glucose level was the best predictor of LVEM.

**Conclusion:**

In this study, deterioration of cardiac function in prediabetic obese children and adolescents was shown. We recommend determining cardiovascular risk and cardiac dysfunction at early stages in prediabetic obese children and adolescents.

**WHAT IS ALREADY KNOWN ON THIS TOPIC?**

Childhood obesity causes subclinical impairment of cardiac function. Left ventricular structural changes have already been demonstrated in obese children.

**WHAT THIS STUDY ADDS?**

Ventricular dysfunction studies have not been performed in obese children and adolescents with prediabetes. Studies have generally been performed in adult population.

## INTRODUCTION

Obesity causes several co-morbidities. Insulin resistance, type 2 diabetes mellitus (T2DM), and cardiovascular impairment are the most important obesity-related complications. When insulin secretion cannot maintain the degree of hyperinsulinemia required to overcome the resistance, prediabetes [impaired glucose tolerance (IGT), impaired fasting glucose] and subsequently T2DM develop (1).

Obesity in children is associated with early structural myocardial disturbances in adulthood. Childhood obesity has been shown to be a cause of subclinical impairment of cardiac function in childhood. Left ventricular structural changes have been demonstrated in obese children and adolescents (2). Increased values for left ventricular mass index (LVMi) and carotid intima-media thickness (c-IMT) as well as abnormal results of Doppler imaging have been reported in childhood obesity (3,4,5,6,7).

Left ventricular dysfunction is a determinant for the development of future heart failure. Tissue Doppler parameters are less load dependent compared to traditional Doppler parameters (3). In particular, markers of ventricular dysfunction, as shown by myocardial tissue Doppler velocities, have not been clearly examined in obese children and adolescents with prediabetes. Studies have generally been performed with the adult population (8).

This study aimed to assess the relationship between prediabetes and ventricular function in obese children and adolescents beyond traditional echocardiographic parameters.

## METHODS

One hundred ninety-eight obese children and adolescents were included in this study. The study was approved by the Necmettin Erbakan University Faculty of Medicine Local Ethics Committee. The boys and girls included in the study were 6 to 18 years of age, free of known diseases, and not taking any medication. Anthropometric parameters were assessed in all patients. Body mass index (BMI) was calculated as weight (in kilograms) divided by height (in meters) squared. Patients with a BMI greater than the 95th percentile for age and gender were considered as obese (9). Waist circumference (WC) was measured at the level of the umbilicus with the patient standing and breathing normally. WC was evaluated using the percentile curves for WC of healthy Turkish children (10). Pubertal development stages were assessed using the Tanner criteria (11,12). Blood pressure was measured with a standard mercury sphygmomanometer. Systolic blood pressure (SBP) and diastolic blood pressure (DBP) values more than the 95^th^ percentile for age, sex, and height were defined as hypertension (13).

After overnight fasting, blood samples were taken for determination of glucose, insulin, total cholesterol, triglyceride, low-density lipoprotein (LDL), and high-density lipoprotein (HDL) cholesterol, and hemoglobin A1c (HbA1c) levels. The homeostasis model assessment of insulin resistance (HOMA-IR; fasting insulinxfasting glucose/22.5) was used as an index of insulin resistance. Insulin resistance was defined as a HOMA-IR of greater than 2.5 in the prepubertal group and greater than 3.16 in the pubertal group (14,15). HOMA2-IR was calculated (16). An oral glucose tolerance test (OGTT) was performed in all subjects with 1.92 g/kg glucose monohydrate and samples taken at 0, 30, 60, 90 and 120 minutes after glucose loading.

Prediabetes was defined according to the American Diabetes Association guidelines (17). Accordingly, impaired fasting plasma glucose was defined as a fasting plasma glucose level of 100 mg/dL to 125 mg/dL, or IGT as shown by a 2-hour plasma glucose of 140 mg/dL to 199 mg/dL in the OGTT, or a HbA1c level between 5.7% and 6.4%.

Echocardiography was done with a Sonos 5500 with a 5.0 MHz transducer in the pediatric cardiology department and the echocardiography data previously done were used in the assessments. All measurements were done according to the criteria defined by the American Society of Echocardiography (18,19). Patients with any congenital or acquired heart disease were discarded from the study group.

LVM was estimated using the formula of Devereux and Reichek (20). The LVMi was calculated by dividing LVM by height2.7 [de Simone et al formula (21)]. Intima-media thickness of the common carotid artery (c-IMT) far wall was measured with the electronic calipers of the machines, as previously described (22).

Tissue Doppler velocities were obtained from three locations in the right and left ventricles. The sample volume was positioned on the lateral aspect of each atrioventricular valve annulus and the basal portion of the interventricular septum. Early (E) diastolic velocities, peak early diastolic myocardial (e’) (LVEM, RVEM), late myocardial velocity (LVAM, RVAM), and peak systolic (s’) (LVSM, RVSM) myocardial velocities were measured by this technique. The E/e’ ratios were calculated (LVEEM, RVEEM) (23).

### Statistical Analysis

Normality was tested. The data were expressed as mean ± standard deviation. Differences were assessed using the Student’s t-test and chi-square test. Correlation and regression analysis were performed. A p-value of <0.05 was accepted to be of statistical significance.

## RESULTS

The prevalence of prediabetes was 40.9% in the obese study population. The mean age was 11.84±2.95 years in prediabetic children and 11.88±2.97 years in non-prediabetic children. 74% of the prediabetic children were pubertal. There was no difference in the presence of prediabetes according to puberty (p=0.82) and sex (p=0.77). There were no statistically significant differences between the two groups with respect to age, gender, or BMI. Traditional risk factors such as SBP, DBP, WC, LDL, total cholesterol, and triglycerides were not statistically different according to prediabetic status. The baseline characteristic features of the two groups are shown in Table 1.

Cardiovascular parameters of patients according to prediabetes are shown in Table 2. The c-IMT difference was not statistically significant (p=0.37), whereas the LVMi was 43.98±10.95 in the prediabetes group and 40.63±10.33 in the non-prediabetes group, findings which were significantly different (p=0.036). LVMi was positively correlated with triglycerides, SBP, WC, and weight standard deviation score, and negatively correlated with HDL cholesterol (p=0.043, r=0.15; p=0.039, r=0.15; p=0.025, r=0.017; p=0.009, r=0.19; p=0.038, r=-0.15, respectively). The statistically significant tissue Doppler findings in prediabetic patients were LVEM and LVEEM (p=0.035, p=0.043, respectively).

In the prediabetes group, LVEM was correlated with fasting glucose (p=0.046) and LVEEM was correlated with SBP (p=0.035). In linear regression analysis, the best predictor of LVEM was fasting glucose in the prediabetes group.

## DISCUSSION

With the increasing prevalence of obesity, there is also an increase in prevalence of T2DM, prediabetes, and insulin resistance in pediatric ages. Effective prevention and treatment of T2DM is already a debate in pediatric populations. Today, the diagnosis of prediabetes and T2DM is increasing, and there is an interest in earlier identification and prevention (24). In Turkey, Kurtoglu et al (25) found the prevalence of prediabetes to be 37% in the boys and 27.8 % in the girls before puberty and 61.7% in the boys and 66.7% in the girls during puberty in obese children and adolescents. In another study, the prevalence of prediabetes was 15.2% in obese adolescents and 25.5% in obese children (1). In other countries, the prevalence of prediabetes in obese adolescents is reported to range from 19% to 39%. In this study, we found a prevalence of 40.6%, a figure similar to previous reports (26).

Studies have recently demonstrated that higher fasting plasma glucose levels within the normoglycemic range might be a predictor of diabetes. Nguyen et al (27) found a significantly increased risk for developing adult prediabetes and T2DM in children in the 86 to 99 mg/dL plasma glucose range after controlling for other traditional cardiometabolic risk factors. In our study, we found fasting glucose levels similar to this in the prediabetes group. There was no significant difference for other cardiometabolic parameters (such as lipid profile, blood pressure). Similar to other studies, our study showed that a high fasting glucose level, a high insulin resistance index, and a high BMI might be potential risk factors for diabetes (27,28).

Tfayli and Arslanian (29) reported that adolescents with T2DM had significantly lower insulin-stimulated total and oxidative glucose disposal, suggesting that a defect in first-phase insulin response is seen early in the development of T2DM in youths, and that defects in second-phase response cause overt diabetes mellitus. IGT or prediabetes is an intermediate phase of altered glucose metabolism that is part of the process of the development of T2DM. Weiss et al (30) mentioned in their manuscript that IGT in obese youth is associated with severe insulin resistance, beta cell dysfunction, and altered abdominal and muscle fat partitioning. In their study, they found that all children who developed T2DM on follow-up had IGT at baseline. Developing diabetes from IGT in adults takes at least 5-10 years. It is speculated that there is an accelerated process in youth. Haemer et al (24) highlighted that insulin levels may be a poor predictor of diabetes risk because while insulin resistance may be accompanied by high insulin levels initially, insulin secretion may decrease later in the progression toward diabetes as a result of glucotoxicity and beta-cell failure In our study, we found both HOMA-IR and insulin level to be significantly different in the prediabetes group.

Studies have shown that prediabetes significantly increases the risk of developing diabetes, but it is reversible. In one study, nearly 50% of severely obese adolescents with prediabetes subsequently reverted to normal glucose tolerance, whereas 24% progressed from prediabetes to diabetes (24). Although the former group appeared to be clinically normal, the effects of the prediabetic state on tissue levels in the cardiovascular system remain unknown.

Obesity and cardiometabolic risk factors have been shown to be associated with vascular changes indicative of early atherosclerosis, or with ventricular hypertrophy, dilatation, and dysfunction. These cardiovascular consequences may be evident in young ages, but childhood obesity is also predictive of similar consequences in adulthood (31).

Several studies have shown that c-IMT is increased in children with cardiovascular risk factors, possibly making it a useful tool for assessing cardiovascular risk in children. Another cardiovascular risk factor is LVM. As shown from the Bogalusa Heart Study, childhood adiposity is related to LVM in adults. Although left ventricular hypertrophy is rare in obese children, cardiac remodeling might be present, which in adults has also been found to predict adverse cardiovascular outcomes (32). In a recent study, Pires et al (32) found both LVMi and c-IMT to be increased in obese children.

Left ventricular hypertrophy is an enlargement related to an increased load on the heart. The most common cause is hypertension. Obesity and prediabetes are additional risk factors for left ventricular hypertrophy (33). In previous studies, increased LVMi was reported in obese patients. With hyperglycemia, glycation end products are produced and irreversible cross-linking with collagen polymers in the myocardial and arterial walls occurs. In this way, myocardial compensation decreases, leading to left ventricular hypertrophy (34). Left ventricular hypertrophy may also reflect neurohormonal and metabolic stimuli causing left ventricular growth. Recent studies showed that LVMi was also increased in children with prediabetes, as in our study (35). c-IMT values were similar to obese patients in the literature but not significantly different from prediabetic patients. c-IMT values were high in obese children and there were variable values for c-IMT in literature. In our study we found c IMT higher according to some studies (36,37). However c-IMT wasn’t statistically different in prediabetes group according to non prediabetes group. This may be due to the small number of patients.

Studies have shown left ventricular diastolic dysfunction to be the first manifestation of myocardial involvement in diabetic patients. The development is multifactorial, including metabolic disturbances, changes in extracellular matrix components, small vessel disease, autonomic dysfunction, and insulin resistance. Therefore, patients with prediabetes may have decreased ventricular function due to prolonged exposure to elevated glucose levels (8,38). Aslan et al (8) showed ventricular impairment in the adult population and suggested detailed evaluation of cardiovascular changes in prediabetic patients.

In a study in prediabetic youth, Shah et al (39) showed deterioration in metabolic profiles with higher BMI z-score, higher SBP, and fasting insulin as well as with increased c-IMT. To our knowledge,there are two studies recently carried out concerning the relationship between prediabetes and cardiac function in obese children and adolescents. Shah et al (39) showed that youth with prediabetes have worse cardiometabolic risk factors and display evidence of increased arterial thickness and stiffness. De Marco et al (35) reported early preclinical systolic and diastolic dysfunction with early cardiovascular alterations also being present in prediabetic adolescents.

In this study, our aim was to confirm that there is a deterioration in ventricular function (not seen in conventional echocardiographic measurements) in prediabetic children,using tissue Doppler echocardiography (39).

Tissue Doppler echocardiography can show subclinical alterations (both the diastolic and systolic impairment) of ventricular functions in obese patients. In one study, obesity-related increased preload volume was considered to be involved in the impairment of diastolic myocardial velocity (7). Harada et al (40) reported that the E/e’ ratio showed the strongest correlation with LV diastolic filling pressure. Van Putte-Katier et al (41) reported a positive correlation between E/e′ ratio and BMI These data suggest that a greater E/e’ ratio is due to impaired ventricular relaxation and is associated with early and subclinical higher ventricular filling pressures in obese young subjects. We speculate that increased glucose levels worsen the function of the left ventricle.

In our data, children with prediabetes were characterized by significantly higher LVEM e’ tissue velocity and higher E to- e’ ratio (LVEEM), compared to non-prediabetic children. Also, early LV diastolic and systolic dysfunction were determined to be present in prediabetic children and adolescents.

Our results indicate that obese prediabetic children are characterized by a higher frequency of increased LVMi and impaired ventricular function. With this study, we also want to emphasize the importance of glucose and blood pressure monitoring in the follow-up of obese children and to state that the assessment of Doppler imaging might be useful in detecting subclinical impairment of cardiac function in prediabetic obese patients at a pediatric age.

## Ethics

Ethics Committee Approval: Necmettin Erbakan University Ethics Committee (Approval number: 2014/572), Informed Consent: Retrospectively designed.

Peer-review: External peer-reviewed.

## Figures and Tables

**Table 1:**
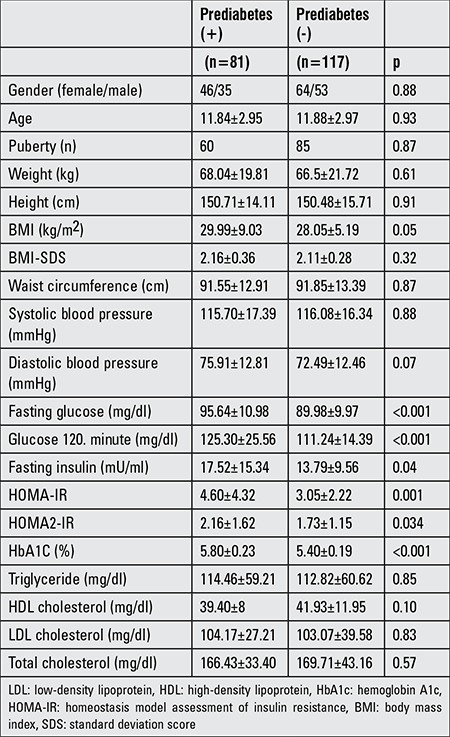
Characteristic features of the prediabetes positive and negative groups

**Table 2:**
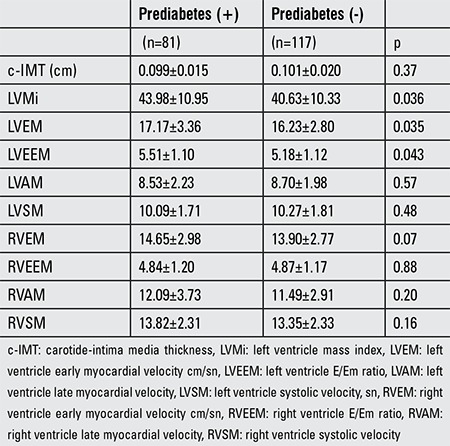
Cardiovascular parameters of patients according to prediabetes
